# Multi-Objective NSGA-II Optimization of Single- and Dual-Fluid ORC–VCC Systems Using Butane and Isobutane

**DOI:** 10.3390/ma17235839

**Published:** 2024-11-28

**Authors:** Łukasz Witanowski

**Affiliations:** Institute of Fluid-Flow Machinery, Polish Academy of Sciences, 80-231 Gdańsk, Poland; lwitanowski@imp.gda.pl

**Keywords:** multi-objective optimization, natural refrigerants, butane, isobutane, waste heat utilization, cooling, turbine, compressor

## Abstract

The urgent need for environmentally sustainable cooling technologies, driven by global regulatory constraints, has intensified the search for natural refrigerants with low global warming potential. This study evaluates the potential of natural refrigerants, specifically butane and isobutane, in advanced single- and dual-fluid Organic Rankine Cycle–Vapor Compression Cycle (ORC–VCC) systems to enhance energy efficiency and environmental sustainability. Using the Non-dominated Sorting Genetic Algorithm II (NSGA-II) within a multi-objective framework, the optimization maximizes key performance metrics such as coefficient of performance (COP) and cooling power, while the Technique for Order Preference by Similarity to Ideal Solution (TOPSIS) method enables a refined ranking of optimal solutions. Findings reveal that the isobutane (ORC)–butane (VCC) dual-fluid configuration achieves the highest overall COP of 0.447 and a cooling capacity of 35.517 kW, surpassing the reference fluid R1233zd, which attains a COP of 0.374 and a cooling capacity of 30.361 kW. Isobutane-based configurations consistently deliver higher COP and cooling capacities than R1233zd, highlighting isobutane’s suitability for applications demanding high energy efficiency. Pressure analysis revealed that R1233zd exhibits the highest pressure ratio of **4.10**, necessitating more complex compressor designs. In contrast, isobutane configurations offer favorable pressure ratios and similar pressure parameters in both single and dual setups, simplifying compressor design requirements. This research provides valuable guidance for developing sustainable ORC–VCC systems by combining effective fluid selection and advanced multi-objective optimization techniques to meet both environmental and operational criteria.

## 1. Introduction

**Figure 1:**
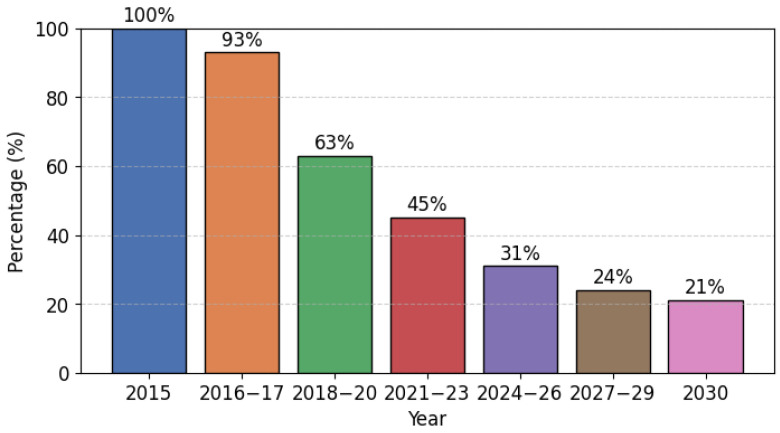
Hydrofluorocarbon phase-down schedule.

The global drive towards environmental sustainability has exerted considerable pressure on the refrigeration and air conditioning industries, particularly in view of the F-Gas Regulation [1]. The regulation stipulates a 79% reduction in the utilization of hydrofluorocarbons by 2030, in comparison to the levels recorded in 2015 (Figure 1). Consequently, there is an urgent need to phase out high global warming potential (GWP) refrigerants. Two main strategies are being adopted to meet these regulatory demands: the introduction of lower GWP refrigerants with reduced system charges, and the use of alternative low-GWP refrigerants approved by equipment manufacturers. Both approaches must carefully balance energy efficiency, system reliability, safety, and economic feasibility.

In addition to these regulatory pressures, there is a growing concern surrounding per- and polyfluoroalkyl substances (PFASs), a group that includes many F-gases. PFAS compounds, which are utilized in closed-loop systems such as heat pumps and the ORC system [2,3], are being subjected to increasing scrutiny due to their persistence in the environment and significant health risks, including carcinogenicity, endocrine disruption, and negative effects on fertility. The European Union’s regulatory efforts, particularly through the REACH restriction, are focused on the reduction of non-polymeric PFASs. However, the long-term degradation of polymeric substances represents an additional risk. In light of their environmental persistence and potential harm, the search for safer alternatives is of great urgency. Natural refrigerants, such as ammonia, butane, carbon dioxide and isobutane, offer a promising solution due to their low global warming potential and lack of ozone depletion potential, making them a safer and more sustainable choice for such systems. However, the deployment of natural refrigerants also introduces novel technical challenges, such as flammability, explosion risks, and the necessity for high-pressure systems or larger equipment. It is essential to address these challenges through careful optimization in order to guarantee safety, efficiency, and cost-effectiveness.

In analyses of ORC–VCC systems [4], a wide range of working fluids have been employed, including those with both high and low GWP and ODP (Ozone Depletion Potential) [5,6,7]. Wang et al. [8] presented a comprehensive theoretical analysis of ORC–VCC system configurations utilizing R245fa as the working fluid. In another study, Karellas et al. [9] conducted an energy–exergy analysis and an economic investigation of an ORC–VCC system in both cogeneration and trigeneration applications, considering three working fluids: R245fa, R152a, and R134a. Nasir et al. [10] expanded this research by exploring seven different working fluids, including butane and isobutane, in both single- and dual-loop systems. The system was distinctive in that it lacked regenerators on either the ORC or VCC side. The analysis was conducted over an external temperature range of 30 °C to 40 °C and a heat source temperature of 100 °C. Two scenarios were optimized at an external temperature of 40 °C, with relative humidity levels of 0% and 50% respectively. In the second scenario, a higher coefficient of performance (COP) defined as the ratio of chilling heat duty to the amount of heat input) of 0.281 was achieved. Another study [11] conducted an intriguing analysis in which the COP was evaluated based on the temperature of the heat source. In this case, 10 working fluids were analyzed in a single-loop system without regenerators. Due to the integrated configuration of both subsystems, it was imperative to maintain a consistent pressure at the outlet of both the compressor and the turbine, which may have contributed to a reduction in overall system efficiency. A comparable methodology, with the same pressure exerted on the turbine and compressor, was employed in a study [12] where three working fluids were evaluated for a single-loop system with a regenerator on the ORC side. The cooling water temperature was 15 °C, and the COP achieved was 0.5. In study [13], an analysis of three working fluids (R1233zd, R1244yd, and R1336mzz) in single and dual configurations was presented. The Non-dominated Sorting Genetic Algorithm III (NSGA-III) optimization method was applied, achieving a maximum COP of 0.57 for the configuration using R1233zd in the ORC and R1244yd in the VCC. Conversely, the highest cooling power was obtained with the configuration using R1244yd in the ORC and R1233zd in the VCC. Table 1 summarizes research conducted on the optimization of ORC–VCC systems. The table provides information on the working fluids utilized, electricity generation, system type (single or dual), and key data related to optimization. Additionally, it includes details regarding the condenser cooling water temperature. The reviewed studies exhibit significant variation in approach. For instance, Zhar et al. [14] used thermal efficiency, exergy efficiency, and levelized cost of energy as objective functions, while Salim et al. [15] focused on cost and thermal efficiency. Patel et al. [16], on the other hand, prioritized cost minimization. Furthermore, the studies differ in the number of decision variables, which range from 5 to 13, and in the condenser cooling water temperature, which varies from 15 °C to 30 °C.

In order to address the existing challenges, this study focuses on the use of natural refrigerants, specifically butane (R-600) and isobutane (R-600a), which offer significant environmental advantages, such as zero ozone depletion potential (ODP) and a very low GWP of 3. Unlike many previous studies, which have not extensively explored the multi-objective optimization of complex systems like ORC–VCC, this research addresses a significant gap in the existing literature. The study employs a multi-objective optimization framework utilizing the NSGA-II algorithm, in conjunction with the Technique for Order Preference by Similarity to Ideal Solution (TOPSIS) for decision-making, to evaluate the performance of small-scale ORC–-VCC systems with butane and isobutane in both single and dual configurations. The optimization process is centered on the maximization of key objectives, including energy efficiency and cooling power. In addition, the results are compared with those obtained using R1233zd as a reference fluid. This integrated approach provides a balanced solution that promotes the development of more sustainable refrigeration and cooling technologies.

## 2. ORC–VCC Model Optimization Methodology

### 2.1. System Description and Parameters

**Figure 2:**
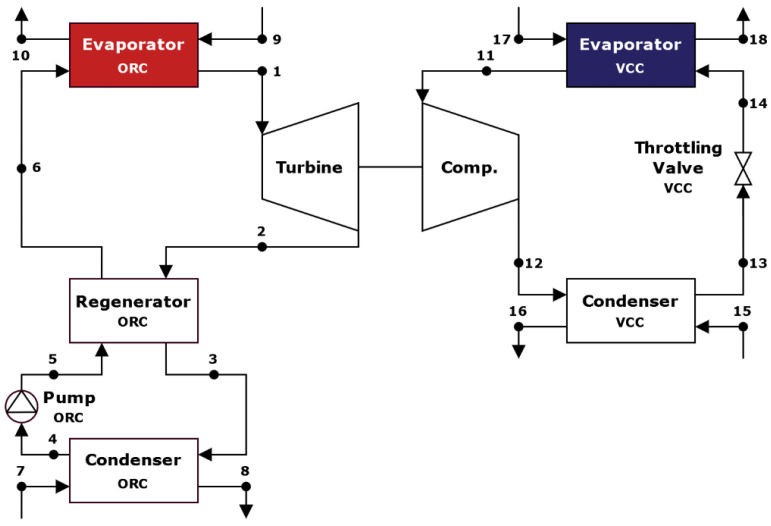
Schematic diagram of the ORC–VCC without electricity generation [12].

**Figure 3:**
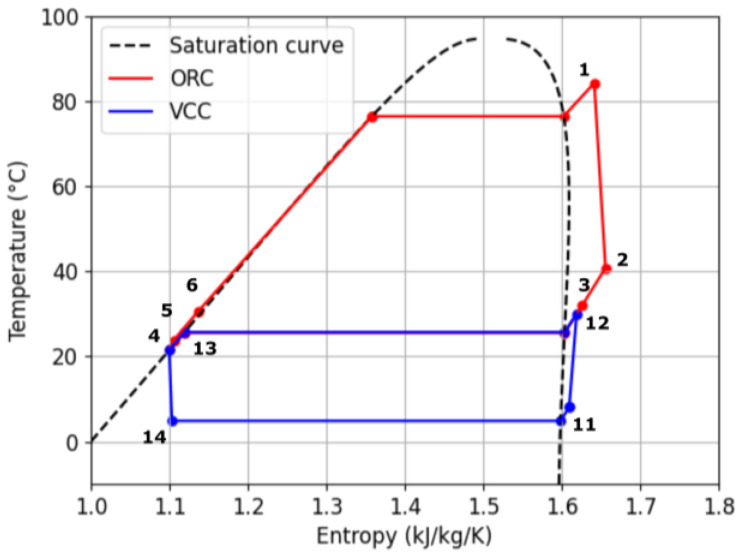
T–s diagram of the ORC–VCC.

**Figure 4:**
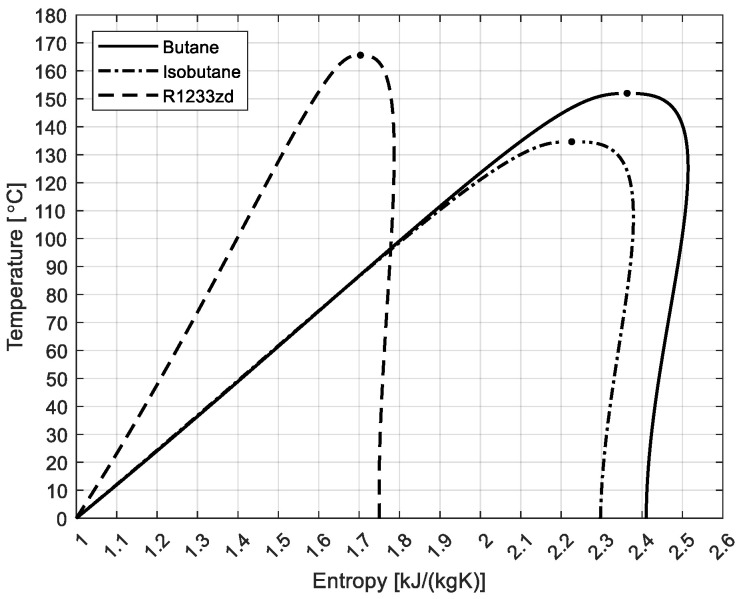
T–s diagram of the analyzed fluids.

The schematic representation of the thermodynamic cycle of the ORC–VCC system is presented in Figure 2 and the temperature–entropy (T–s) diagram in Figure 3. The system comprises an ORC with a regenerator subsystem and a VCC subsystem. The potential benefits of employing both a single and a dual working fluid were investigated as part of the analytical process. In the initial configuration, a single refrigerant is employed throughout the cycle. In contrast, the second solution utilizes two distinct refrigerants, one in the ORC cycle and another in the VCC cycle. The T–s diagram shown in Figure 4 illustrates the thermodynamic properties of three analyzed working fluids: butane, isobutane, and R1233zd [20,21]. Each curve represents the phase change boundaries and critical points of these fluids, providing insight into their suitability for use in ORC–VCC systems. Butane has a critical temperature of 151.98 °C, pressure of 37.96 bar, and density of 228 kg/m^3^, with a molar mass of 58.122 kg/kmol. Isobutane, with the similar molar mass as butane, has a slightly lower critical temperature (134.66 °C) and pressure (36.29 bar), with a critical density of 225.5 kg/m^3^. R1233zd has a much higher molar mass (130.5 kg/kmol) and critical temperature (166.45 °C), with a density of 480.23 kg/m^3^.

### 2.2. Thermodynamic Model

The thermodynamic analysis of the ORC–VCC was carried out using Python 3.10.2 and NIST REFPROP 10 [20,21]. The latter was employed to calculate the thermodynamic properties of the working fluids through a cycle calculation function implemented in the aforementioned programming language. In order to simplify the model of the ORC–VCC, the following assumptions were made:

- Steady-state conditions are maintained by the system.
- The temperature and mass flow rate of the heat source are assumed to be maintained at a constant level.
- Pressure drops and energy losses in the heat exchangers, pipelines, and valves are considered negligible.
- The efficiencies of the turbine, compressor, and feed pump are assumed to remain constant and unaffected by operating conditions.
- The work produced by the ORC expander was intended solely to meet the work requirements of the VCC compressor.
- The process through the throttling valve of the VCC was assumed to be isenthalpic.
- The power of the auxiliary systems, including the heat removal system from the condensers (encompassing the power of the pump and the cooling tower fan) and the heat supply systems to the evaporators, was not considered.

The numerical assumptions related to the system are detailed in Table 2. The efficiencies of the machines were selected with a significant safety margin, with the internal efficiencies of both the compressor and turbine set at 80% in accordance with conservative estimates. In experimental studies of the ORC–VCC system conducted by Grauberger et al. [22], comparable turbine and compressor efficiency values were observed.

The thermodynamic process was constructed in accordance with the basic ORC with regenerator and VCC cycles. In order to determine a heat flux delivered to the cycle in the ORC evaporator, the following equation is applied:

(1) $$Q_{orc\_evaporator}=m_{9}(h_{9}-h_{10})$$

The quantity of heat supplied to the VCC evaporator is thus determined by the following equation:

(2) $$Q_{vcc\_evaporator}=m_{17}(h_{17}-h_{18})$$

Consequently, the coefficient of performance is given by:

(3) $$COP=\frac{Q_{vcc\_evaporator}}{Q_{orc\_evaporator}}$$

To determine the net efficiency of the ORC cycle, the power consumed by the feed pump and the power generated by the turbine must first be calculated. Subsequently, the cycle efficiency is obtained using the heat supplied to the evaporator of the ORC system:

(4) $$h_{5s-4}=h_{5s}-h_{4}$$

(5) $$h_{5-4}=\frac{h_{5s-4}}{\eta_{p}}$$

(6) $$P_{p}=m_{1}h_{5-4}$$

(7) $$h_{1-2s}=h_{1}-h_{2s}$$

(8) $$h_{1-2}=h_{1s-2s}\eta_{t}$$

(9) $$P_{t}=m_{1}h_{1-2}$$

(10) $$\eta_{ORC}=\frac{P_{t}-P_{p}}{Q_{orc\_evaporator}}$$

The refrigeration COP (COP_VCC_) is calculated by dividing the heat supplied by the evaporator by the power consumed by the compressor. To achieve this, it is first necessary to calculate the isentropic drop in the compressor, the actual drop (taking into account the efficiency of the compressor), and the compressor power. These calculations are then used to calculate the COP_VCC_:

(11) $$h_{12s-11}=h_{12s}-h_{11}$$

(12) $$h_{12-11}=\frac{h_{12s-11}}{\eta_{c}}$$

(13) $$P_{c}=m_{11}h_{12-11}$$

(14) $${COP}_{VCC}=\frac{Q_{vcc\_evaporator}}{P_{c}}$$

### 2.3. Optimization Procedure and Algorithm

The optimization methodology employed in this study incorporates advanced multi-objective optimization techniques, utilizing the pymoo library 0.6.1.3 [28], which is a robust framework for Python 3.10.2 designed for multi-objective optimization. This is integrated with a code developed in-house which accurately models and evaluates the performance of a small-scale ORC–VCC system. This combination allows for precise and efficient exploration of the solution space, effectively addressing the complexity of the system’s design parameters and objectives. The ORC–VCC system is modeled using custom-built code, which simulates the thermodynamic behavior of the system. This enables a comprehensive evaluation of key performance indicators, which in turn provides valuable insight into the optimization process. The problem is formulated with 13 decision variables, each of which directly influences the system’s behavior and is bounded within specified limits (lower bounds ranging from 0.1 to 50, and upper bounds from 5 to 85). These variables capture essential parameters that dictate the system’s overall performance (Table 3). In addition to the optimization process, the handling of data represents a crucial aspect of this study. The pandas library 2.0.3 [29] was employed for data cleaning and preprocessing, thereby ensuring that the datasets used in the optimization process were accurate and well structured. The utilization of NumPy 1.24.2 [30] facilitated the data analysis and processing tasks, providing efficient numerical computations. Matplotlib 3.9.2 [31] was employed for the generation of detailed plots and graphs, which aided in the clear presentation of optimization results and performance metrics. This integrated approach, which employed pandas, NumPy, and Matplotlib, ensured the data-driven insights were effectively analyzed and visualized, thereby supporting the optimization process by revealing critical patterns and trade-offs within the data.

The optimization process targets two primary objectives:

- Objective Function 1: Maximizing the COP, a critical measure of the system’s efficiency in converting energy input into cooling output.
  (15) $$f_{1}=COP$$
- Objective Function 2: Maximizing the cooling power, ensuring that the system can deliver the highest possible cooling capacity.
  (16) $${f_{2}=Q}_{vcc\_evaporator}$$

The optimization tasks are executed using the pymoo library, which was specifically chosen for its powerful capabilities in handling multi-objective optimization problems. pymoo supports a range of evolutionary algorithms, including the NSGA-III [32,33] used in this study. To further assist in the decision-making process, the TOPSIS [34,35] method was employed. This method enables a comparative analysis of the optimized solutions by ranking them based on their proximity to an ideal solution, thus facilitating a more comprehensive evaluation of trade-offs between competing performance metrics. By integrating TOPSIS with the optimization approach, the study ensures a robust selection of solutions that balance efficiency and cooling power.

The application of multi-objective optimization in conjunction with the TOPSIS method represents an efficacious approach to the selection of optimal solutions in the context of complex scientific problems [36,37,38]. Jankowski et al. [39] employed this method to undertake a multi-objective optimization of an ORC system in conjunction with a one-dimensional radial-inflow turbine. The optimal point was identified through a TOPSIS analysis of the variables SP (size parameter) and EPC (electricity production cost). In the decision-making process, priority was assigned to the economic performance of the system, with a weight of w1 = 0.7 assigned to the EPC and w2 = 0.3 assigned to the SP. The objective function values at the TOPSIS-determined optimal point were 0.09 USD/kWh for EPC and 0.040 for SP. Sadeghi et al. [40] developed a fuzzy optimization model to address the inherent conflicts between competing objectives in agricultural water supply projects, with a particular focus on the optimization of time, cost, quality, and resource leveling. The model was validated using the NSGA-II and Multi-objective Particle Swarm Optimization, and subsequently applied to a real project. The fuzzy TOPSIS method identified an optimal schedule with a 178-day duration, which met the employer’s time requirement and achieved 82.13% of the expected quality. The project cost was 9175 million Rials, with the resource leveling objective scoring 2260, reflecting a balanced approach to stakeholder priorities.

The optimization process is initiated by defining the problem through the class, wherein the bespoke code for ORC–VCC system calculations is embedded within the evaluation function. This function is executed during each iteration of the optimization to assess the performance of each candidate solution in accordance with the three objectives. The study employs a systematic examination of nine cases, each defined by a distinct combination of selected refrigerants:

- a_1_: Butane (ORC and VCC).
- a_2_: Isobutane (ORC and VCC).
- a_3_: Butane (ORC), Isobutane (VCC).
- a_4_: Isobutane (ORC), Butane (VCC).
- a_5_: R1233zd (ORC and VCC).

These cases were designed to explore the performance impacts of different refrigerant combinations on the system’s objectives. Each case was optimized separately, providing a comprehensive comparison across different fluid pairings. For each case, the NSGA-II algorithm within pymoo is configured with a population size of 65. The algorithm uses reference directions generated by the Das–Dennis method, ensuring a well-distributed search across the three-dimensional objective space. The optimization runs for 100 generations, during which the population is refined to converge on a set of non-dominated solutions that offer the best trade-offs between maximizing COP and cooling power.

The combination of the pymoo library with a tailored ORC–VCC system modeling approach offers a robust and adaptable framework for this study. Pymoo’s capabilities in handling multi-objective problems make it an ideal choice, particularly given the complex, non-linear relationships between the decision variables and the objectives. The custom code ensures that the thermodynamic and operational characteristics of the ORC–VCC system are accurately captured, allowing for precise evaluations of each candidate solution. This integrated approach allows for a thorough exploration of the design space, leading to the identification of refrigerant combinations and system configurations that optimize the critical objectives. The findings are expected to provide valuable insights for the design and operational strategies of ORC–VCC systems in energy recovery and cooling applications.

## 3. Results

A total of 32,500 outputs were generated during the optimization process across five independent optimization runs. Following data cleansing, 16,513 outputs were retained for further analysis, with a particular focus on 29 key variables. A range of system configurations and parameter settings were explored through these optimizations, providing valuable insights into the behavior and performance of the ORC–VCC system under varying conditions. This analysis facilitated the identification of optimal configurations, contributing to a deeper understanding of the system’s operational efficiency and environmental impact.

**Figure 5:**
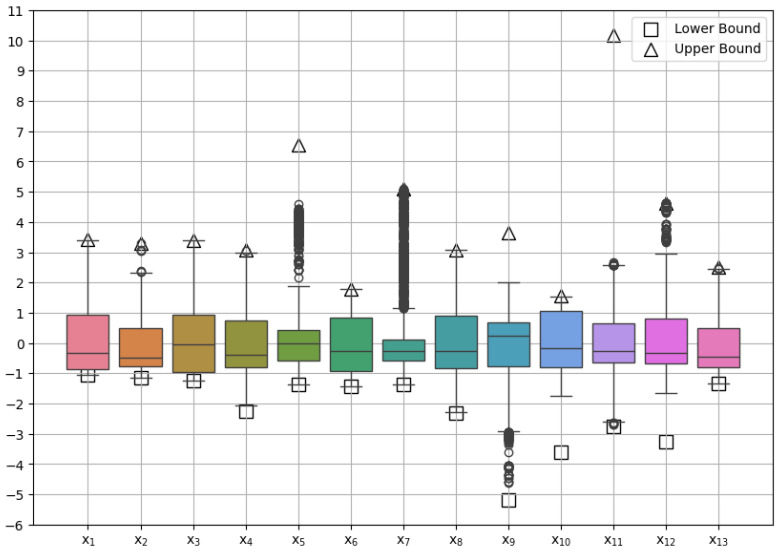
The distribution of decision variables in the optimization runs.

In Figure 5, a boxplot is provided to visually represent the distribution of 13 key variables, labeled x_1_ to x_13_, within the standardized dataset; each box encapsulates the interquartile range (IQR), serving as a measure of variability in the data by reflecting the spread between the 25th and 75th percentiles. This interquartile range highlights the central tendency and dispersion, minimizing the influence of extreme values. The median, representing the midpoint of the dataset, is displayed and remains resistant to outliers, which helps to capture the underlying distribution more accurately. The whiskers, extending to 1.5 times the IQR, indicate the primary range where most data points reside, while any points outside this range are considered outliers, represented by individual dots. Additional markers denote the original standardized lower and upper bounds for each variable, with squares for lower bounds and triangles for upper bounds, offering context for how far actual values deviate from initial design constraints. This visualization reveals patterns essential for understanding the optimization space and algorithm’s exploration behavior; for example, variables x_5_, x_7_, x_9_, and x_12_ display a wide distribution with numerous outliers, suggesting that a broad range of values was sampled. Outliers in these variables indicate areas where the system either reached feasible solution limits or encountered less typical scenarios. Moreover, the standardized bound markers provide insight into the extent of exploration; in several cases, such as x_1_, values approached boundaries, indicating thorough examination of extreme regions in the parameter space. However, not all variables followed this pattern; for instance, x_5_ and x_9_ did not reach their upper bounds, likely due to boundary-adjacent values being excluded during optimization, which may reflect a tendency to favor solutions away from extremes. This behavior implies that, in balancing multiple objectives, the algorithm may have been constrained by factors discouraging the selection of upper-bound values, thus favoring more conservative areas within the search space. These insights are critical for understanding the algorithm’s overall performance and the nature of solutions favored during the optimization process.

**Figure 6:**
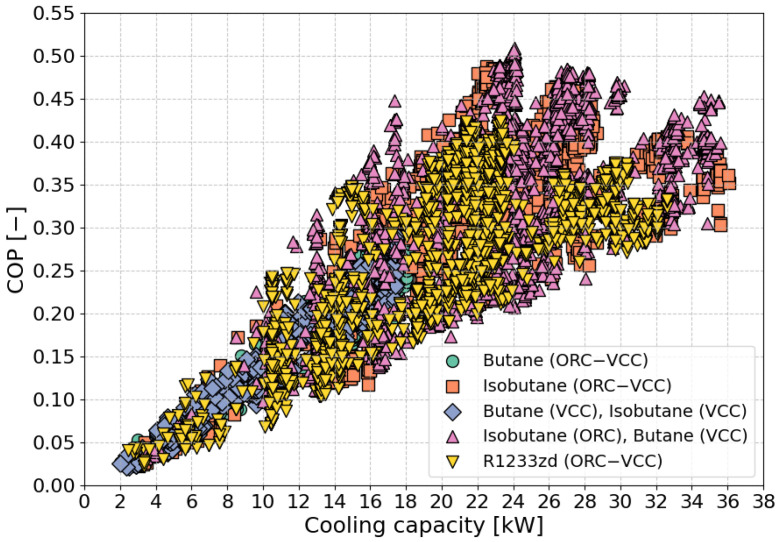
Distribution of COP vs. cooling capacity for various optimization scenarios.

Figure 6 illustrates the correlation between cooling capacity (in kW) and the COP for various fluid combinations within the ORC–VCC system. At the lower end of the plot, a minimal cooling capacity of approximately 2 kW is observed, accompanied by a low COP around 0.02, with this region primarily dominated by butane-based configurations within the ORC–VCC system. As cooling capacity increases, a noticeable upward trend in COP emerges, indicating a positive correlation between the two variables. The maximum cooling capacity, observed at around 38 kW, is achieved by fluids such as R1233zd (ORC–VCC), isobutane (ORC–VCC), and the isobutane (ORC)–butane (VCC) combination, where COP values range from 0.25 to 0.45—relatively high compared to the lower-capacity regions. These fluids exhibit a broader performance range, suggesting efficiency across varying conditions, with high COP values reflecting their effectiveness in converting input energy into useful cooling power, making them well suited for high-performance systems. In contrast, for lower cooling capacities, particularly below 10 kW, COP values generally remain below 0.2, with fewer instances exceeding this threshold. As cooling capacity surpasses 20 kW, both COP values and data point density increase, indicating that higher cooling capacities correspond with enhanced COP performance, especially when optimized fluid combinations like R1233zd and isobutane are employed. This trend suggests that systems operating at higher capacities deliver more efficient cooling, making them ideal for applications requiring high-performance efficiency.

**Figure 7:**
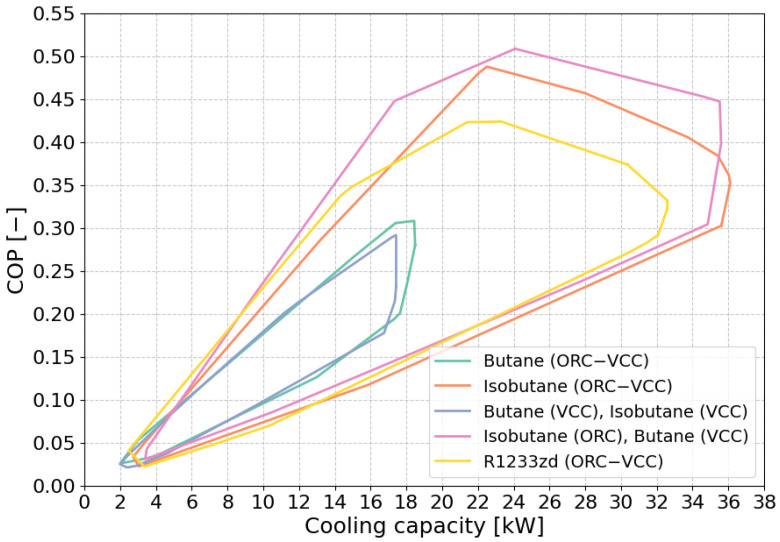
Performance boundaries of COP vs. cooling capacity for different optimization scenarios.

The performance boundaries of the COP as a function of cooling capacity for different fluid combinations are illustrated in Figure 7. The plot demonstrates that R1233zd (ORC–VCC), isobutane (ORC–VCC), and isobutane (ORC)–butane (VCC) exhibit the widest range of performance, with cooling capacities extending up to 38 kW and COP values reaching approximately 0.5. These fluids demonstrate high efficiency across a wide range of operating conditions, making them well suited for higher capacity applications. In contrast, the performance boundaries for butane (ORC–VCC) and the butane (ORC)–isobutane (VCC) combination are more limited, with cooling capacities not exceeding 18 kW and maximum COP values around 0.25. These narrower boundaries suggest that these configurations may be less suitable for high-capacity systems, possibly due to thermodynamic constraints or specific design limitations. The plot further illustrates that as cooling capacity increases, most fluid combinations show a corresponding rise in COP, indicating improved efficiency. However, the decline in COP at higher capacities for butane-based systems suggests potential limitations when using these fluids in larger systems.

**Figure 8:**
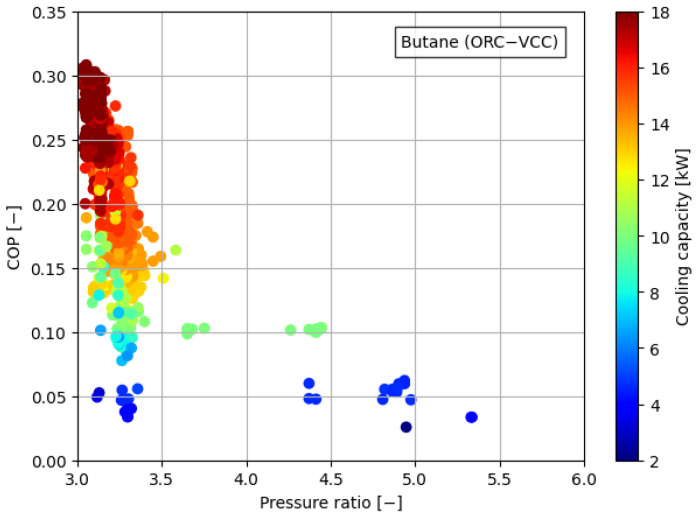
Relationship between COP, pressure ratio, and cooling generation for butane.

**Figure 9:**
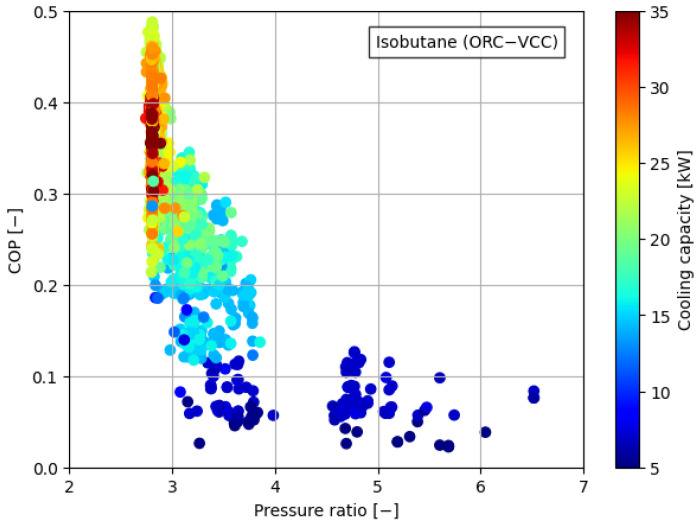
Relationship between COP, pressure ratio, and cooling generation for isobutane.

**Figure 10:**
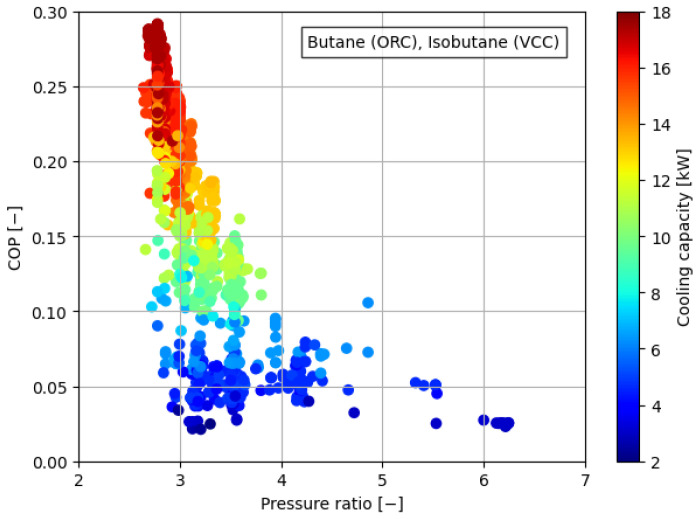
Relationship between COP, pressure ratio, and cooling generation for butane in the ORC cycle and isobutane in the VCC cycle.

**Figure 11:**
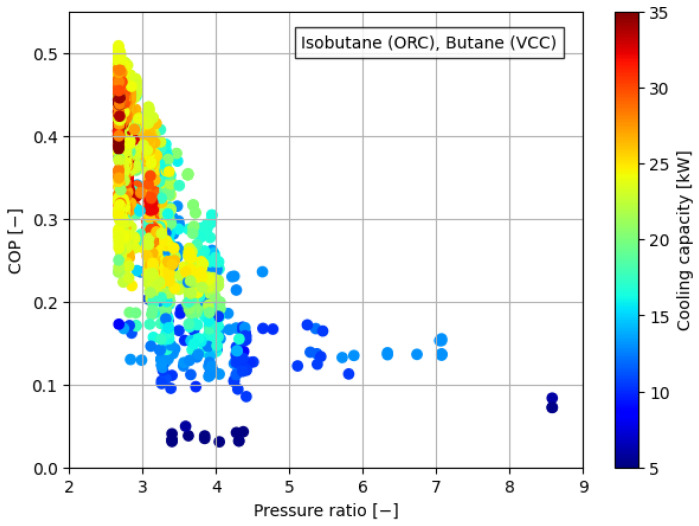
Relationship between COP, pressure ratio, and cooling generation for isobutane in the ORC cycle and butane in the VCC cycle.

**Figure 12:**
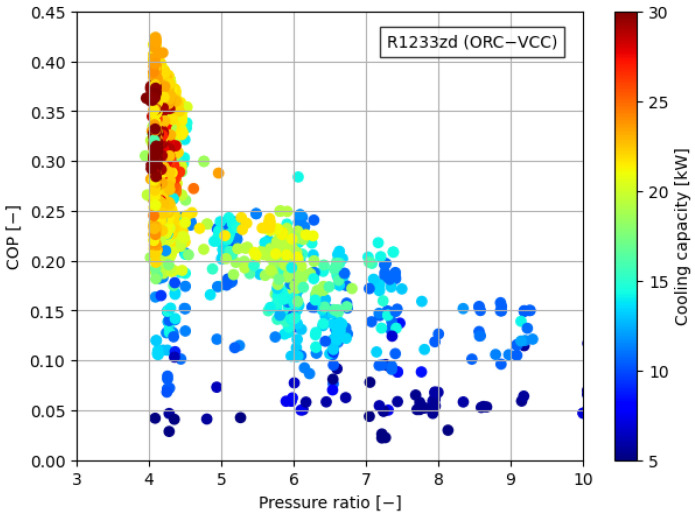
Relationship between COP, pressure ratio, and cooling generation for R1233zd.

Figure 8, Figure 9, Figure 10, Figure 11 and Figure 12 illustrate the relationships between COP and pressure ratio. For all the analyzed working fluids, higher COP values were observed at lower pressure ratios. The lowest pressure ratios in the compressor were identified in systems where isobutane was used in the VCC system, as well as in the dual system comprising isobutane (ORC) and butane (VCC). In these cases, pressure ratios dropped below 3. In the reference case, the lowest pressure ratios reached a level of 4. It is important to note that in all cases, a decrease in COP was accompanied by a reduction in cooling capacity, coinciding with an increase in the pressure ratio. The reduction in COP with an increasing pressure ratio can be attributed to the rise in the enthalpy difference between the compressor outlet and inlet. This occurs concurrently with a decrease in the enthalpy difference within the evaporator, which diminishes the heat absorption capacity of the system. The combined effect of higher compressor work and reduced evaporator performance leads to a decline in overall system efficiency.

In Table 4, the optimal parameter points for each characteristic in the ORC–VCC system are presented, with a focus on maximizing four key criteria: COP, Q_vcc_evaporator_, ORC netto efficiency (η_ORC_) and COP_VCC_. Each row corresponds to a specific configuration, labeled as cases a_1_ through a_5_, which are differentiated by working fluid selections for the ORC and VCC components. The COP is used as the primary indicator of cooling performance, which is essential for assessing the efficiency of the cooling application in the ORC–VCC system. In conventional refrigeration units, a COP formula similar to COP_VCC_ is typically applied, which depends on electric input. However, due to the waste heat-based operation of this system, a unique COP formulation has been adopted to capture its performance under waste heat-driven conditions. The η_ORC_ parameter is introduced specifically to evaluate the performance of the ORC component within the combined ORC–VCC system. Performance trade-offs across configurations are highlighted in the table, with the maximum COP reaching 0.509 in case a_4_. The maximum Q_vcc_evaporator_ is observed in case a_2_, with a value of 36.109, while the highest η_ORC_ value of 7.963 is achieved in case a_5_. Notably, similar η_ORC_ values exceeding 7% are achieved in cases a_2_ and a_4_, indicating consistently high ORC performance in these configurations. Although COP_VCC_ reaches its peak of 6.664 in case a_4_, a slightly lower COP_VCC_ of 6.219 is observed in case a_3_.

**Figure 13:**
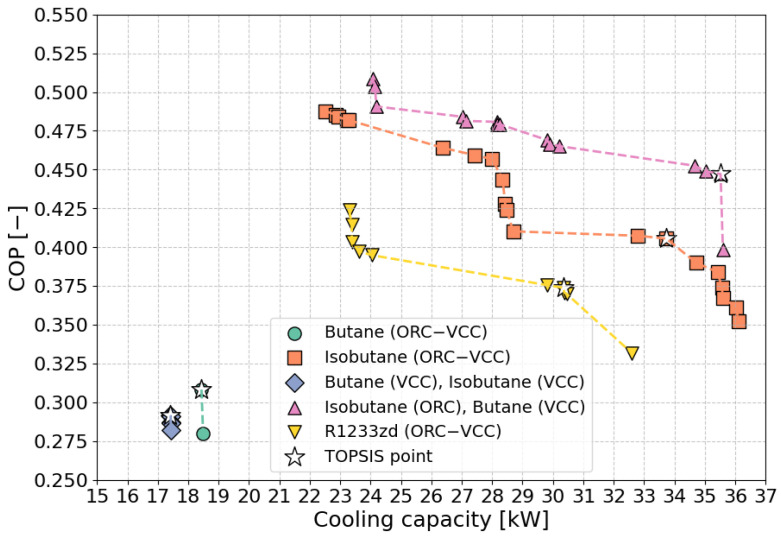
Pareto frontier with TOPSIS-selected optimal points for COP (weight = 0.5) vs. cooling capacity (weight = 0.5).

**Figure 14:**
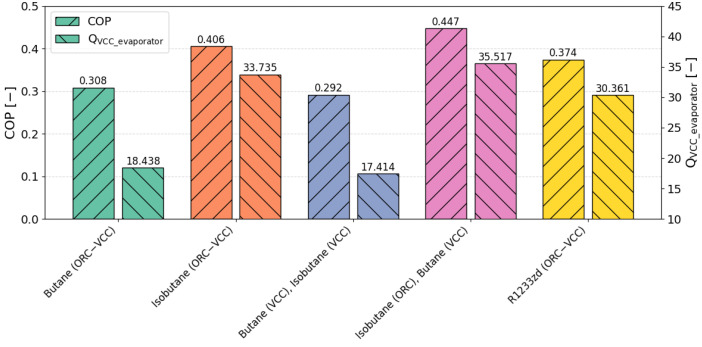
Comparison of COP and cooling capacity (Q_VCC_evaporator_) for TOPSIS-selected optimal points (COP weight = 0.5, cooling capacity weight = 0.5).

In Figure 13 the Pareto front is presented for different ORC–VCC configurations, illustrating the trade-off between COP and cooling capacity, both given equal importance (weight = 0.5), a weighting choice selected for further analysis. The working fluids analyzed include butane, isobutane, and R1233zd, used in various configurations, with each symbol representing a different fluid combination within the ORC and VCC systems. The TOPSIS-selected optimal points, marked with stars, represent configurations that best balance COP and cooling capacity, with COP values for these points ranging from 0.28 to 0.50 and cooling capacities spanning 17 kW to 35 kW. The selected TOPSIS points guide the selection of ORC–VCC configurations, offering a compromise between efficiency and cooling output, which is essential for applications requiring efficient waste heat recovery and robust cooling performance. In reference to Figure 13, Figure 14 was prepared to present a comparison of the coefficient of performance (COP) and cooling capacity for TOPSIS-selected optimal points. The highest COP, reaching 0.447, is observed for the isobutane (ORC)–butane (VCC) configuration, paired with a cooling capacity of 35.517 kW. Meanwhile, the reference fluid, R1233zd, (ORC–VCC) achieves a COP of 0.374 and a cooling capacity of 30.361 kW. Notably, isobutane-based configurations achieve higher COP and cooling capacity values than the reference fluid, R1233zd.

**Figure 15:**
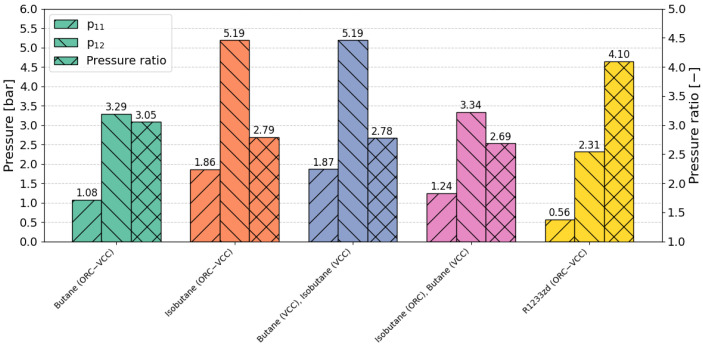
Comparison of pressures (p_11_ and p_12_) and pressure ratio for TOPSIS-selected optimal points (COP weight = 0.5, cooling capacity weight = 0.5).

In Figure 15 a comparison of the pressures p_11_, p_12_, and pressure ratio is presented. The reference fluid, R1233zd, operates under a unique condition, with an inlet compressor pressure below 1 bar, placing it in a vacuum range. This vacuum operation could pose challenges related to pressure maintenance and potential leaks, impacting the system’s reliability and operational integrity. Additionally, R1233zd is characterized by the highest pressure ratio among the fluids examined, reaching a value of 4.10, which suggests that more complex compressor construction may be required to handle the increased number of stages. For configurations where isobutane is used in the VCC cycle, whether in single or dual configurations, the pressure parameters are nearly identical. In contrast, butane configurations show notable differences; in the dual configuration, the pressure ratio is more than 0.3 lower compared to the single configuration. The difference between butane and isobutane configurations is likely due to the lower power output achieved in the butane ORC cycle, which impacts the VCC cycle parameters by limiting its operational range. This comparison underscores the significant variability in pressure characteristics across different fluid configurations, illustrating how design choices impact performance and operational feasibility within the ORC–VCC system.

**Figure 16:**
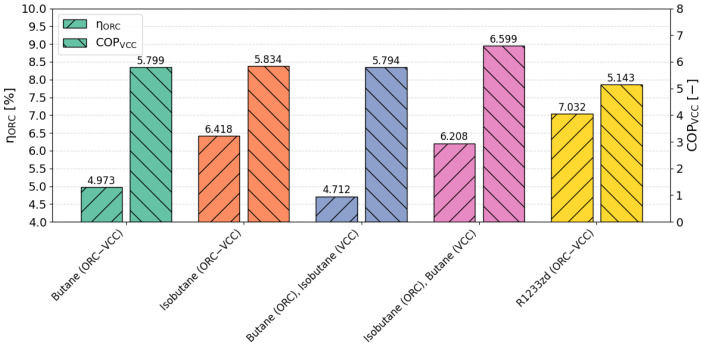
Comparison of η_ORC_ and COP_VCC_ for TOPSIS-selected optimal points (COP weight = 0.5, cooling capacity weight = 0.5).

Figure 16 presents a comparison of η_ORC_ and COP_VCC_. Across the selected configurations, η_ORC_ ranges from a minimum of 4.71% to a maximum of 7.03%, while COP_VCC_ varies between 5.143 and 6.599. The reference fluid, R1233zd, achieves the highest value for η_ORC_ (7.03%) and the lowest value for COP_VCC_ (5.143), highlighting that efficiency is not necessarily correlated with achieving the highest value in all cases, particularly within the ORC–VCC system. However, these values do not correlate with the results in Figure 8, where R1233zd ranks third in terms of COP performance, highlighting a divergence in efficiency metrics. The configuration with isobutane in the ORC cycle and butane in the VCC cycle exhibits the highest refrigeration COP (6.599) and the third η_ORC_ (4.71%). Despite this, it achieves the highest overall COP in Figure 8, demonstrating its effectiveness in maximizing cooling performance. These results illustrate the importance of analyzing key cycle parameters and, even more critically, selecting them appropriately when constructing the objective function in the optimization process. Not every indicator needs to reach its maximum value for the overall system to perform optimally, emphasizing the need for a balanced approach in multi-criteria optimization.

**Figure 17:**
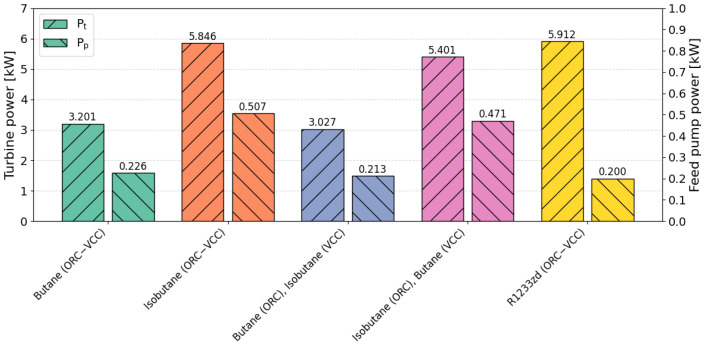
Comparison of turbine power and feed pump power for TOPSIS-selected optimal points (COP weight = 0.5, cooling capacity weight = 0.5).

A critical aspect of conventional refrigeration systems is the power demand required for operation, specifically the electrical energy necessary to operate the compressor. In the analyzed ORC–VCC system, the turbine is solely responsible for supplying the required power to the compressor, without generating additional electrical energy. As a result, the only component requiring electrical energy input is the feed pump of the ORC system. Figure 17 presents the turbine and feed pump power for selected cases. In systems utilizing isobutane in the ORC system, as well as R1233zd, the turbine power exceeds 5 kW. However, in systems employing isobutane, the pump power is significantly higher, reaching up to twice the power of the reference system. This increase is due to the need to maintain elevated pressure differences in ORC systems operating with isobutane.

**Figure 18:**
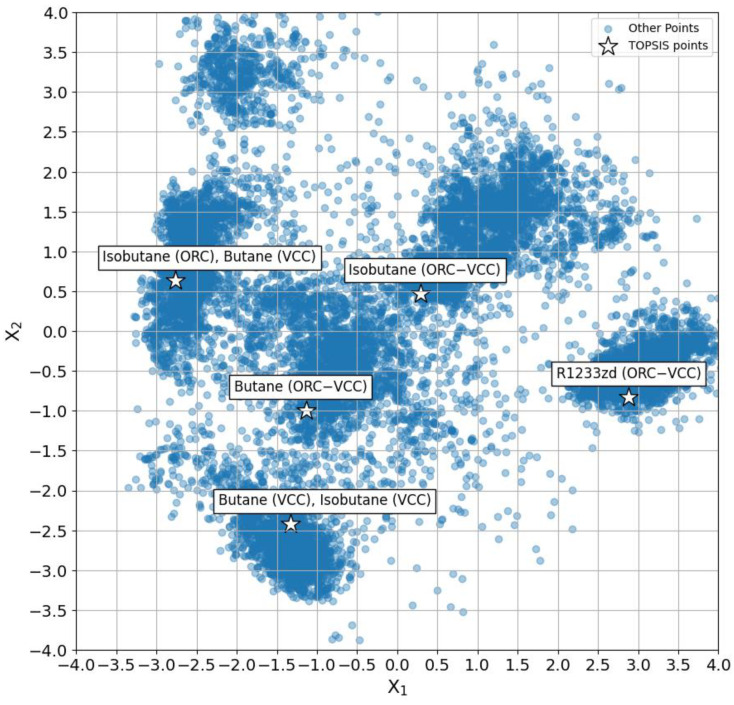
PCA distribution of analyzed scenarios with highlighted TOPSIS-selected optimal points.

In Figure 18, the distribution of analyzed scenarios within the ORC–VCC system is presented through a Principal Component Analysis (PCA) plot, highlighting the spread across two principal components, X_1_ and X_2_. The data points represent various fluid combinations and configurations examined for their cooling capacity and COP under different conditions. TOPSIS-selected optimal points, determined with equal weights of 0.5 for COP and cooling capacity, are marked with stars. These highlighted points correspond to specific fluid configurations. The spatial clustering of these points within the PCA plot indicates the variability in performance and design attributes, with each cluster representing regions where distinct fluid combinations achieve similar trade-offs between efficiency and cooling capacity. Notably, the close grouping of fluids like butane and isobutane suggests similarities in design requirements for these fluids, particularly under configurations targeting moderate cooling capacity and COP values. In contrast, the distinct separation of R1233zd (ORC–VCC) from the other clusters indicates a design approach with unique parameters tailored to achieve optimal performance with this fluid, likely due to differences in thermodynamic properties and compatibility with high-capacity requirements. The positioning of TOPSIS points within or near these clusters suggests their alignment with optimal performance ranges, capturing configurations that balance efficiency and output in the ORC–VCC system.

**Figure 19:**
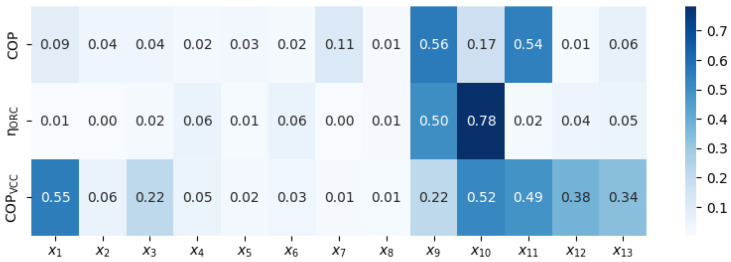
Summary of significance of the decision variables on the cycle parameters.

In Figure 19, the significance of various decision variables (x_1_ through x_13_) on key cycle parameters is summarized through a heat map, where the influence on the COP, η_ORC_, and COP_VCC_ is illustrated. Darker shades are used to indicate a stronger correlation between each decision variable and the cycle parameters. It is observed that x_9_ and x_10_ exert the most substantial influence on η_ORC_, with correlation values reaching up to 0.50 and 0.78, respectively. Lower correlation values observed for other variables indicate a limited impact on cycle performance, implying that adjustments to these parameters may have minimal influence on key parameters. This visualization provides insight into which variables can be prioritized or adjusted to achieve desired performance outcomes.

**Figure 20:**
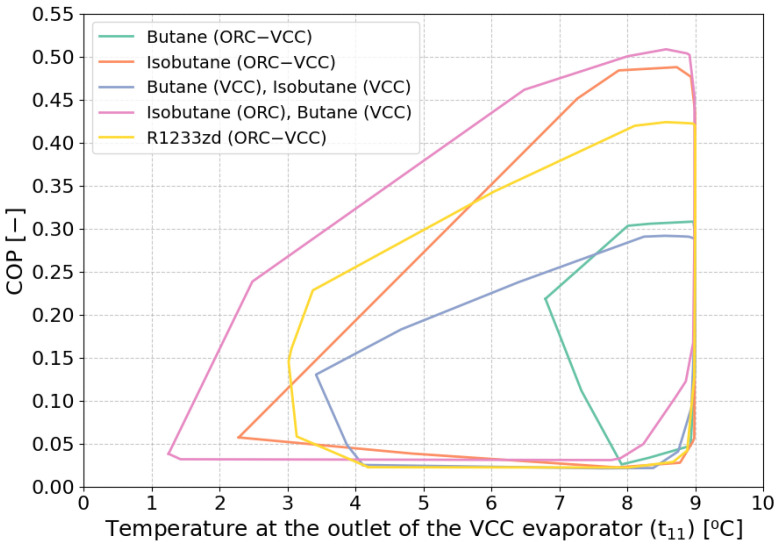
Performance boundaries of COP vs. temperature at the outlet of the VCC evaporator for different optimization scenarios.

A significant factor influencing the characteristic parameters of the ORC–VCC system is the outlet temperature of the VCC evaporator. Figure 20 illustrates the COP curve as a function of the evaporator outlet temperature. In general, an increase in the evaporator outlet temperature leads to higher COP values. However, the observed decrease in COP at elevated temperatures indicates changes in operating parameters, such as a notable increase in the pressure ratio, which has an adverse effect on COP.

## 4. Conclusions

This work explores, through an optimization approach, the potential of using natural working fluids in advanced single- and dual-fluid ORC–VCC systems, resulting in superior energy efficiency and enhanced environmental sustainability. For both the optimal COP values and those derived from the TOPSIS analysis, isobutane achieves higher performance than R1233zd, which is crucial when selecting a working fluid for various operational requirements of the system. The highest COP value was achieved with the dual configuration, highlighting its potential for enhanced efficiency. Additionally, this analysis illustrates the performance differences between single and dual configurations, suggesting that dual configurations may offer distinct advantages in terms of energy output and efficiency. Moreover, the pressure ratios achieved with isobutane are more favorable compared to R1233zd, which can further support its selection. Additionally, the minimum operating pressure, which is higher for isobutane, could also be a deciding factor in the final choice of fluid, as it simplifies pressure maintenance and reduces the risk of vacuum conditions. It is important to note that key parameters of the ORC and VCC cycles, such as ORC efficiency and refrigeration COP, do not directly reflect the overall COP of the combined ORC–VCC system, emphasizing the complexity of optimizing a dual-cycle system as a cohesive whole.

In light of the findings of this study, further research is planned with the objective of enhancing the knowledge base in this area. Future work will concentrate on incorporating dynamic operating conditions in order to evaluate system performance under fluctuating temperatures of the heat source and sink. A principal objective of this research is the incorporation of comprehensive turbocompressor characteristics into the dynamic analysis, thereby facilitating a more precise evaluation of transient behavior and operational stability under diverse loads. This approach will also facilitate the development of advanced control strategies that are specifically tailored to the behavior of turbocompressors within the ORC–VCC system. Furthermore, environmental and economic analyses, including life cycle assessment and cost–benefit analysis, will be incorporated to provide a more comprehensive understanding of the system’s viability and sustainability.

## Figures and Tables

**Table 1:** Summary of works on ORC–VCC optimization.

Author	Working Fluid	Electricity Production	Single/Dual Fluid	Optimization Information	Cooling Water Temperature
Zhar et al. [14]	R123, R11, R113	No	Single	Multi-objective; thermal efficiency, exergy efficiency, and levelized cost of energy optimized; 5 decision variables.	-
Salim et al. [15]	R245fa, R245ca, R235ea, R410A	No	Dual	Multi-objective; cost and thermal efficiency optimized; 12, scenarios; 6 decision variables.	25 °C
Patel et al. [16]	n-Pentane, R410a	Yes	Dual	Single objective; total annual cost function optimized; 6 decision variables.	27 °C
Mounier et al. [17]	R134a, R152a, R245fa, R600a	No	Single	Multi-objective; exergetic efficiency and total cost optimized.	30 °C
Bao et al. [18]	R1270, R1234yf, R290, R161, R1234ze, R152a	No	Both	Single objective; second-law efficiency optimized; 4 scenarios (different system configurations); 11 decision variables.	25 °C
Nasir et al. [10]	R134a, Isobutane	No	Dual	Single objective; COP optimized; 2 scenarios, 3 decision variables.	-
Witanowski [12]	R1233zd, R1336mzz, R1224yd	No	Single	Single and multi-objective; IRR, COP, and NPV optimized; 6 scenarios; 11 decision variables.	15 °C
Witanowski [13]	R1233zd, R1336mzz, R1224yd	No	Both	Multi-objective; COP, cooling power, and pressure ratio optimized; 9 scenarios (single and dual configurations), 13 decision variables.	25 °C
Witanowski [19]	R1233zd, R1336mzz, R1224yd, R1234yf, R1234zez	Yes	Single	Multi-objective; COP and power netto optimized; 10 scenarios (different cooling temperatures), 13 decision variables.	15 °C, 30 °C
Witanowski [this work]	Butane, Isobutane, R1233zd	No	Both	Multi-objective; TOPSIS; COP and cooling power; 5 scenarios (single and dual configurations), 13 decision variables.	25 °C

**Table 2:** Efficiency and thermodynamic assumptions of the ORC–VCC system.

Parameter	Symbol	Unit	Value
Chilled water temperature (cold side)	T_18_	°C	8
Chilled water temperature (hot side)	T_17_	°C	12
Cooling water temperature (cold side)	T_7_, T_15_	°C	25
Heat source mass flow	m_9_	kg/s	1
Heat source temperature (hot side)	T_9_	°C	90
Compressor efficiency (VCC)	η_c_	-	80% [23,24]
Feed pump efficiency (ORC)	η_p_	-	50% [25]
Turbine efficiency (ORC)	η_t_	-	80% [26,27]

**Table 3:** Decision variables and their boundaries for the ORC–VCC system optimization.

Arg.	Parameter	Symbol	Unit	Lower Bounds	Upper Bounds
x_1_	Evaporator pinch temperature difference (VCC)	ΔT_vcc_ev_	K	3	10
x_2_	Degree of superheating in evaporator (VCC)	ΔT_vcc_sup_	K	3	10
x_3_	Degree of subcooling in condenser (VCC)	ΔT_vcc_sub_	K	3	10
x_4_	Regenerator pinch temperature difference (ORC)	ΔT_orc_reg_	K	3	15
x_5_	Degree of superheating in evaporator (ORC)	ΔT_orc_sup_	K	3	15
x_6_	Degree of subcooling in condenser (ORC)	ΔT_orc_sub_	K	7	15
x_7_	Evaporator pinch temperature difference (ORC)	ΔT_orc_ev_	K	3	10
x_8_	Condenser pinch temperature difference (ORC)	ΔT_orc_con_	K	3	10
x_9_	Saturation temperature in evaporator (ORC)	T_1sv_	°C	50	85
x_10_	Saturation temperature in condenser (ORC)	T_4sv_	°C	25	55
x_11_	Chilled water mass flow rate (VCC)	m_16_	kg/s	0.1	5
x_12_	Pressure on the inlet of the compressor (VCC)	p_12_	bar	0.1	10
x_13_	Condenser pinch temperature difference (VCC)	ΔT_vcc_con_	K	3	10

**Table 4:** The best points for each characteristic parameter.

Parameter	Case	COP	Q_vcc_evap_	η_ORC_	COP_VCC_
Unit	[–]	[–]	[kW]	[%]	[–]
Max COP	a_1_	0.308	18.438	4.973	5.799
	a_2_	0.488	22.504	7.636	5.795
	a_3_	0.292	17.414	4.712	5.794
	a_4_	0.509	24.080	7.224	6.632
	a_5_	0.424	23.296	7.963	5.166
Max Q_vcc_evap_	a_1_	0.280	18.491	4.662	5.793
	a_2_	0.352	36.109	5.751	5.810
	a_3_	0.282	17.429	4.633	5.777
	a_4_	0.398	35.595	5.498	6.627
	a_5_	0.330	32.591	6.216	5.194
Max η_ORC_	a_1_	0.218	12.983	4.983	5.286
	a_2_	0.480	22.019	7.651	5.815
	a_3_	0.288	17.372	4.871	5.778
	a_4_	0.458	23.230	7.297	6.273
	a_5_	0.424	23.296	7.963	5.166
Max COP_VCC_	a_1_	0.301	17.309	4.979	5.851
	a_2_	0.382	31.624	6.218	5.930
	a_3_	0.249	16.055	4.348	6.219
	a_4_	0.482	23.239	6.89	6.664
	a_5_	0.305	18.539	6.746	5.346

## Data Availability

The original contributions presented in the study are included in the article; further inquiries can be directed to the corresponding author.
